# Whole genome sequencing in pharmacogenomics

**DOI:** 10.3389/fphar.2015.00061

**Published:** 2015-03-26

**Authors:** Theodora Katsila, George P. Patrinos

**Affiliations:** Department of Pharmacy, School of Health Sciences, University of PatrasPatras, Greec

**Keywords:** pharmacogenomics, whole genome sequencing, pharmacogenomics biomarkers, genome-wide association studies, genotype, pesonalized medicine

## Abstract

Pharmacogenomics aims to shed light on the role of genes and genomic variants in clinical treatment response. Although, several drug–gene relationships are characterized to date, many challenges still remain toward the application of pharmacogenomics in the clinic; clinical guidelines for pharmacogenomic testing are still in their infancy, whereas the emerging high throughput genotyping technologies produce a tsunami of new findings. Herein, the potential of whole genome sequencing on pharmacogenomics research and clinical application are highlighted.

## Introduction

Pharmacogenomics (PGx) aims to develop strategies for individualizing therapy to optimize drug efficacy and minimize toxicity on the basis of our improved understanding of how genomic variants influence drug response. PGx research focuses on both pharmacodynamic and pharmacokinetic effects (Crews et al., 2012). Pharmacodynamic-PGx effects correspond to differences in patient response due to genomic variants in drug target pathways – or even, the drug targets themselves. Pharmacokinetic-PGx effects refer to differences in patient response because of genomic variants in the pathways involved in drug metabolism or processing.

Pharmacogenomics research has exhibited a profound acceleration during the past decades and the discovery and investigation of gene variants have followed several paths (Squassina et al., 2010). This was made possible either by employing medical records (Neuraz et al., 2013), or adopting candidate gene and genome-wide association approaches (Wang and Weinshilboum, 2008; Motsinger-Reif et al., 2013). Undoubtedly, the advent of next generation sequencing (NGS) has created unprecedented opportunities towards the analysis of whole genomes, obtaining a full picture of people’s variomes. A variome refers to the set of genetic variations in populations of a single species that have been acquired in a relatively short time on an evolutionary scale (Pavlopoulos et al., 2013). To date, whole exome and/or whole genome sequencing can be easily performed using several commercially available or proprietary platforms exhibiting a high degree of accuracy and at a reasonable cost (Nekrutenko and Taylor, 2012).

## Whole Genome Sequencing and PGx Challenges

Obtaining a full picture of a person’s variome is necessary, particularly in the case of novel unique variants that may either render a drug metabolizing enzyme and/or transporter inactive, or disable its expression and, hence, the carrier of the variant intermediate or even poor metabolizer. NGS approaches are gradually being adopted for PGx, involving either whole exome (Price et al., 2012) or whole genome sequencing (Meyerson et al., 2010). For detecting DNA variants, whole exome sequencing is indeed a remarkable advance, yet an interim strategy, since, even if perfect technical accuracy is assumed, the exome comprises about 1% of the entire genome (Feero, 2014). Although several PGx effects are caused by amino-acid substitutions, PGx variants do not necessarily occur within coding regions and in some cases, not even within the genes themselves. Instead, it is well established that variants that affect transcript splicing or regulation of transcription are also associated to PGx. Furthermore, population based association studies have given rise to variants that may not be causative, but can be statistically associated with the causative marker(s) (Ardlie et al., 2002). To further complicate our choices, whole exome sequencing is currently more cost-effective, but current capture probes can only target known exons, while regulatory and un-translated regions are not sequenced. Additionally, there is a significant bias (target enrichment step); capture probes’ efficiency varies considerably and hence, some sequences fail to be targeted. Finally, the commercial target enrichment kits vary considerably and as such, not all templates are sequenced with equal efficiency. Consequently, a significant proportion of variants may go undetected (Gamazon et al., 2012; Altman et al., 2013).

There are very few whole genome sequencing studies in PGx, despite the fact that genetic disorders have already begun to greatly benefit from whole genome sequencing applications. Ashley et al. (2010) revealed gene variants suggestive of clopidogrel resistance and positive response to lipid-lowering therapy, indicating the need for a low initial dose for warfarin. In 2011, a substantial amount of novel/uncharacterized variations (predicted to alter protein function) were obtained by whole genome re-sequencing (Drögemöller et al., 2011). In another study that involved 14,002 individuals, Nelson et al. (2012) explored rare genetic variants by sequencing 202 genes encoding drug targets and concluded that human populations harbor an abundance of rare variants, many of which are deleterious and relate to disease risk. Drögemöller et al. (2013) performed a critical analysis on the unmet needs of PGx research on schizophrenia showing that there is a percentage of “inaccessible genome,” including the CYP and HLA genes. Recently, whole genome sequencing was exploited toward the identification of novel and putatively causative genomic variants, affecting the structure and function of 231 pharmacogenes in a large number of human genomes from various ethnic backgrounds (Mizzi et al., 2014). In the same study, the personalized PGx profiles of a seven-member family of Greek origin were defined and then, delineated with the anticoagulation treatment response observed in two family members.

## Whole Genome Sequencing for PGx in the Clinic

Recent evidence, although limited at the present time, supports the idea that whole genome sequencing is capable of revealing unique (or rare) PGx markers that would otherwise go undetected, if conventional genetic screening methods were employed.

Today, much of the challenges of whole genome sequencing to become clinically viable lie in (i) the costs of sequencing technologies, (ii) the regulation scheme regarding the use of (next-generation) sequencers as medical devices, (iii) setting up a (centralized) whole genome sequencing facility, and (iv) training clinicians to interpret PGx data (Kampourakis et al., 2014; Mooney, 2014). The current cost of whole genome sequencing by which a comprehensive personalized PGx profile will be obtained, including almost all of the germline and *de novo* genomic variants needed to manage all current and future treatment modalities lies in the range of US$3000 and is declining. Hence, it is anticipated that soon its cost effectiveness will be appreciated, when compared to the costs of testing for a single or several markers in few pharmacogenes (from US$300 up to US$1500, respectively; Fragoulakis et al., 2015). Recently, the Illumina MiSeqDx sequencer was authorized by FDA (Sheridan, 2014), although the sequencing coverage is particularly low (read length in paired end run of 2°×°150 bp and hence, a 20× coverage for a 50 Mb exome). This event raises great expectations toward the impact of PGx tests in the clinic. While regulatory challenges are still playing an evolving role in the application of PGx testing, sample outsourcing for data analysis, and interpretation might be the answer to the obstacles of setting up a centralized whole genome sequencing facility and clinicians’ training. Ultimately, it will only be a matter of time until testing cost reimbursement is adopted by national insurance bodies (Fragoulakis et al., 2015).

In light of the above, design and implementation of advanced informatics solutions that ease to fill-in the gap between PGx research findings and clinical practice emerges as a major need. In 2014, major – although, limited – milestones have been set. eMERGE-PGx aims to (i) deploy PGRNseq, assessing sequence variation in 84 pharmacogenes in 9,000 patients and several clinical sites, (ii) integrate clinically validated PGx genotypes into electronic health records (EHRs) with associated clinical decision support, and (iii) develop a PGx variants’ repository of unknown significance linked to a repository of EHRs-based clinical phenotype data for on-going PGx discovery (Rasmussen-Torvik et al., 2014). Similar work was undertaken by our group, which presented the development of an integrated electronic ‘PGx assistant,’ designed to provide personalized drug recommendations based upon linked genotype-to-phenotype PGx data and support biomedical researchers in the identification of PGx-related gene variants (Potamias et al., 2014), while the delineation of genomic variants with rare drug outcomes is nowadays an emerging research question in PGx and several groups and international consortia are gradually engaging to investigate this interplay (see www.genomicmedicinealliance.org). The latter would only be possible through a whole genome sequencing approach.

## Conclusion and Future Perspectives

As whole genome and/or whole exome sequencing approaches begin to take hold in clinical care, not only how sequencing technologies evolve, but also how they get integrated into a clinical setting are of utmost importance regarding the development of PGx clinical tests and the understanding of the genetic effects on a prescription and treatment level that need to become more readily available to the clinician. So far, the translation of PGx research findings into clinical practice has been slow. The advent of whole genome sequencing technology will offer an outstanding potential toward the clinical application of PGx. Some key components regarding successful clinical implementation have been already addressed and actions have been already taken, whereas others are to be met.

## Conflict of Interest Statement

The authors declare that the research was conducted in the absence of any commercial or financial relationships that could be construed as a potential conflict of interest.
